# Journalism

**Published:** 1883-10

**Authors:** 


### EDITORIAL.

### Journalism.

The future of dental journalism is a subject that ought to interest every truly professional dentist in the country.

Its beginning, in 1839, in the establishment of the "*American Journal of Dental Science*," was an epoch in the history of the profession, marking a beginning of progress that before had not been dreamed of. There were many doubts as to the probabilities of the success of a monthly journal, of the proportions it then assumed, devoted wholly to dentistry. Such doubts, however were soon removed, for within five years after, the "*Dental Register of the West*," a quarterly journal, was established. Not many years after this the "*Dental News Letter*" and "*Dental Recorder*," were projected and issued; and from that time to the present new periodicals have appeared; till now twenty or more exist—eight or ten would perhaps embrace the more important of them.

Some have originated from a necessity for additional means of communication, and record of the views, opinions, and suggestions of members of the profession, as well as a means of marking the steps of progress. Others have been projected and sustained as a means of conveying to the profession a knowledge of the production and sale of materials, instruments, &c., used by the profession.

Notwithstanding, occasional criticism, the dental journals of the country will compare as a whole, with other professional journals, but this is, perhaps, not enough. They ought to be better, and would be, if all who are interested, and all who ought to be, would do what they could for their improvement and elevation. But change in this respect is being made.

Another question in reference to our journalism is worthy of some consideration, and that is the feasibility of the different journals assuming each, in the main, a particular branch to which special attention would be given. For instance one might be devoted chiefly to the subject of education, students, office pupilage, colleges, and college instruction, &c. Another to association work—encouraging or aiding the formation and sustenance of societies, publishing their proceedings, and making a record of the important work of all dental societies in the country.

Another devote special attention to prosthetic dentistry. Another to operative dentistry. Another to dental pathology and therapeutics.

These are only named for illustration; upon the adoption of such a plan, other subjects would be suggested. By some such division of attention and labor, the work on each particular division would be better accomplished, than is possible in the present diffusive method; each subject would be more thoroughly studied and extensively canvassed than now, and would thus assume a new interest. Our journals then, instead of coming in competition with each other, would simply be parts of a grand whole, each interested in the other. An arrangement of this sort would not be without some precedent. The American Medical Association has recently established the "Journal of the American Medical Association."

The British Dental Association has its monthly journal devoted chiefly to society matters.

Would it not be an indication of progress if some of our jour nals should adopt the course here suggested?

